# 2313: A path perspective on bio-psychosocial predictors of health status in peripheral arterial disease

**DOI:** 10.1017/cts.2017.258

**Published:** 2018-05-10

**Authors:** Nikhil Satchidanand, Jeffrey Fine, Gregory S. Cherr

## Abstract

OBJECTIVES/SPECIFIC AIMS: To explore associations among bio-psychosocial factors predictive of overall physical and mental health status as assessed using the SF-12 Health Survey. METHODS/STUDY POPULATION: Community-dwelling, male and female elders with peripheral arterial disease (PAD) were administered an assessment battery to identify factors associated with self-assessed physical and mental health status using the SF-12 Health Survey. The battery included an assessment of pain, depressive symptoms, perceived social support, perceived psychological stress, physical function, as well as selected demographic variables. RESULTS/ANTICIPATED RESULTS: Preliminary linear regression analyses have identified several factors predictive of physical and mental health status including depressive symptoms, pain, perceived stress, and physical function. A more in-depth examination using path analysis is anticipated to reveal important mediational associations, wherein physical function is a strong mediator between bio-psychosocial factors and overall physical and mental health status. DISCUSSION/SIGNIFICANCE OF IMPACT: Aging is often associated with a reduction in physical and mental well-being, frequently exacerbated by the development and progression of chronic disease. PAD is a common chronic condition that places significant burden on the older patient and their family. Identifying and developing a more in-depth understanding of the factors that impact health status in PAD is an important and timely objective. We anticipate that our findings will inform development of more targeted and effective intervention strategies we can employ to improve the quality of life among elders struggling to manage PAD.

